# Long-Term and Short-Term Forecasting of Oriental Fruit Moth (*Grapholita molesta*) Trap Catches from Apple Orchards in South Korea Using Time Series Models

**DOI:** 10.3390/plants15040624

**Published:** 2026-02-16

**Authors:** Steven Kim, Seong Heo

**Affiliations:** 1 Department of Mathematics and Statistics, California State University, Monterey Bay, Seaside, CA 93955, USA; stkim@csumb.edu; 2 Department of Horticulture, Kongju National University, Yesan 32439, Republic of Korea

**Keywords:** apple, oriental fruit moth, Prophet, SARIMA, VAR

## Abstract

The oriental fruit moth (OFM), also known as *Grapholita molesta*, is a major agricultural pest causing significant economic loss of apple growers in South Korea. This study demonstrates the application of time series models for describing the national and regional patterns of OFM occurrences in the last decade and for forecasting future OFM occurrences. The seasonal autoregression integrated moving average (SARIMA), Prophet, and vector autoregressive (VAR) models are compared for both long- and short-term predictions. The analysis shows that short-term predictions are more reliable than long-term predictions for the number of OMF trap catches, and the multivariate time series model does not necessarily provide better predictive performance with province-level aggregated data. Though the Prophet and VAR model fits bimonthly province-level data better than the SARIMA model, the VAR model shows poor predictive performance, and the SARIMA model showed as or more reliable predictions than the Prophet model in this study. This study presents both the potential and challenges for establishing a Smart Integrated Pest Management (IPM) system capable of monitoring and predicting OFM occurrences and implementing regional pest control strategies. The usefulness of time series analysis can be leveraged by frequent orchard-level data reporting, pest management records, and precise local environment information.

## 1. Introduction

The oriental fruit moth (OFM), also known as *Grapholita molesta*, originated in Northeast Asia, and has been currently spread worldwide including America, Europe, and Australia. The OFM is a major pest of fruit trees, and it damages new shoots and fruits of apples, pears, peaches, and other fruits [1,2]. The pests overwinter as larvae in racks of rough tree bark, and the larvae infest and bore into young shoots and fruit trees [3]. In South Korea, infestations on peaches, plums, and other stone fruits began to increase markedly in the 1980s [4,5,6], but there were insignificant damages caused by the OFM until the early 1990s [5]. Pome fruit crops were severely damaged in the late 1990s, especially in commercially cultivated pear orchards which were poorly managed [7,8], and similar cases were observed in apple trees [2,9]. The damage inflicted by the OFM is categorized into two types. The first type occurs before the fruit sets. Pests infest new shoots and branches, and those tissues wilt and break eventually. In the second type, larvae migrate from leaves to the fruit, penetrating into the pericarp near the fruit stalk and feeding on it. The resulting larval feeding promotes viscous exudation, which may cause secondary fungal rot.

Researchers have monitored and managed the OFM by using sex pheromone traps and chemical controls [6]. For pest species associated with apples, past studies focused on population dynamics models and the management technologies began as early as in the 1970s and 1980s in Michigan [10,11]. The population dynamics models for major apple pests, such as codling moth (*Cydia pomonella*) and OFM (*Grapholita molesta*), have been further developed and implemented in fruit orchards. The population dynamics models have been useful for forecasting the emergence of the first generation and pest management interventions [11,12]. More sophisticated predictive models have been developed for field-monitoring data gathered by sex pheromone traps, and they have been used to forecast the occurrence of OFM in practice [3,13,14,15,16]. These models predict the cumulative proportion of insects emerging during the spring generation based on the accumulated degree-days exceeding 8.14 °C from January 1 in a given year.

Researchers in South Korea have observed four generations of these insects per year [7,17]. According to their observations, the first generation reaches the peak in late April to early May; the second generation peaks around mid-June; the third generation peaks in late July to early August; and the fourth generation peaks in late August to early September. As aforementioned, existing models have been primarily developed for predicting the emergence timing of first-generation pests, not for forecasting the subsequent generation pests. The first-generation pests do not significantly contribute to economic losses due to their feeding habits on shoots and branches. For farmers, pest control during the fruit-feeding stages (second to fourth generation) is of greater importance than controlling infestations on young shoots. The exact occurrence timing and population size of the subsequent generations are volatile, and pest outbreaks fluctuate depending on habitat, climate conditions, and other unobserved factors.

A recent study used two time series models, the Prophet and the seasonal autoregressive integrated moving average (SARIMA), for describing and forecasting the number of OFM trap catches in South Korean peach orchards [18]. In that study, the Prophet model fitted observed data points and predicted future data points better than the SARIMA model. The time series decomposition by the Prophet model reasonably described seasonal outbreaks of OFM, and it showed that the annual number of outbreaks (peaks) varied by time and region. The predictive performance (forecasting one year ahead) also varied by region, resulting from a negative R^2^ value to an R^2^ value of 0.53. To improve the forecasting of the OFM emergence, we have two new considerations in this study. The first consideration is the use of a vector autoregression model which utilizes multivariate time series simultaneously, instead of modeling univariate time series separately. If the number of OFM trap catches in one region is strongly correlated with other regions, we can expect that a multivariate time series model can improve the predictive performance. The second consideration is short-term prediction. It is very challenging to forecast the number of OFM trap catches to be observed in the subsequent year. As time series data are accumulated sequentially, a time series model can be updated as soon as a new data point is available, and it can forecast the next time point. Assuming that data points closer in time are more correlated than data points further in time, the short-term predictions are expected to be more reliable than the long-term predictions. This study attempted time series analysis by applying the above models on OFM trap catches in South Korean apple orchards and compared OFM occurrences at both the national and province levels for spatial analysis.

The overarching goal of this study is to establish a scientific foundation for developing a Smart Integrated Pest Management (IPM) system that integrates real-time monitoring with predictive analytics. Specifically, this study aims to achieve three practical objectives: (1) to evaluate whether time series models can provide reliable forecasts for supporting timely pest control decisions at the national and provincial levels, (2) to evaluate short-term predictions versus long-term predictions for operational pest management, and (3) to identify regional differences in OFM occurrence patterns that may require differentiated management strategies.

This research article is structured as follows. The results obtained from the time series models are presented first in Section 2, and the results are discussed in Section 3. The data source and quality, trapping protocol, and statistical analysis are described in Section 4. Readers may read Section 4 prior to Section 2 for details about the data acquisition, materials, and statistical analysis prior to the results. Finally, the concluding statements are in Section 5.

## 2. Results

This section presents the results from the three time series models described in Section 4.2, and is structured as follows. The time series decomposition, model fits, and forecasts of the national average data are presented in Section 2.1. As practitioners make decisions and take actions appropriate for each province in South Korea, analyzing province-level data may be more practical than the national average data. The results of the province of GB, which cultivates most apples in the Korean Peninsula, are presented in Section 2.2, and the results of the province of JB are presented in Section 2.3. We show that short-term forecasts are generally better than long-term forecasts, and the predictive performance is summarized and compared in Section 2.4.

### 2.1. National Average Data

**Figure 1:**
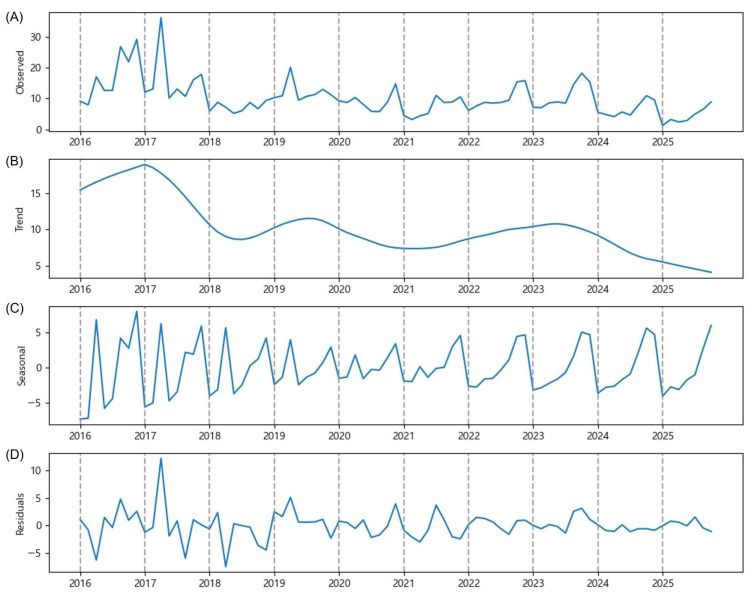
Time series decomposition of the number of OFM trap catches (y-axis) observed bimonthly between June and September from 2016 to 2025. They are the national average data: (**A**) the observed number of OFM trap catches, (**B**) estimated trend component, (**C**) estimated seasonal component, and (**D**) residuals.

Figure 1 presents the time series decomposition estimated by the Prophet. The highest national average number of OFM trap catches (36.1) was observed on 1 July 2017 (Figure 1A). The time series forecasting is accurate when the trend component and the seasonal component show clear patterns with large magnitude relative to the residual component. The trend component clearly shows a decreasing pattern, from an average of fifteen trap catches to an average of five trap catches over the last decade (Figure 1B). The seasonal component, with a magnitude mostly between −5 and +5, shows double-peak patterns until 2021, where the first peak occurred in July and the second peak occurred in September, and single-peak patterns since 2022, where the peak occurred in September (Figure 1C). After explaining the observed time series by the trend and seasonal components, the magnitude of residuals (random noise) is mostly between −5 and +5, which is smaller than the magnitude of the trend and seasonal components combined. The time series decomposition indicates that the life cycle of OFM is not simply random, and the number of OFM trap catches is predictable to some degree.

**Figure 2:**
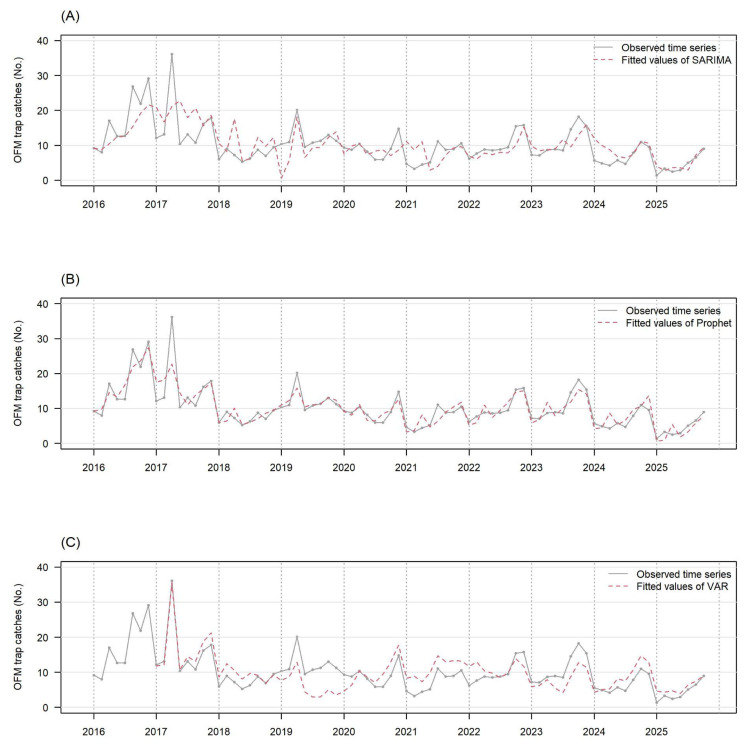
The observed number of OFM trap catches (gray solid line) and the fitted value by each time series model (red dotted line) are presented in this figure. They are the national average data. The fitted values are calculated by the SARIMA model (panel (**A**)), the Prophet model (panel (**B**)), and the VAR model (panel (**C**)). The VAR model needs observed data of the previous year for fitted values, so fitted values of 2016 are not available.

Figure 2A–C present the observed number of OFM trap catches (gray solid line) and the fitted values by the SARIMA-BIC, Prophet, and VAR (red dotted line), respectively. These fitted values were obtained by fitting all observed data points from 2016 to 2025. The VAR model exactly fitted the historical record on 1 September 2017, and the three models described the subsequent outbreaks that occurred in September fairly well, except for a few occasions (e.g., the substantially underestimated peak on 1 September 2019 by VAR). The respective R^2^ values were 0.411, 0.795, and 0.635 for the SARIMA-BIC, Prophet, and VAR (Table 1) which implies that the Prophet and VAR models fitted the national average number of OFM trap catches better than the SARIMA did.

Supplementary Figure S1A–C presents the same observed time series data from 2021 to 2025 and the long-term predictions by the three models. Unlike the fitted values in Figure 2A–C, the outcomes were severely overpredicted by all three models for 2024. The respective R^2^ values for the long-term predictions were 0.177, 0.285, and <0 for the SARIMA-BIC, Prophet, and VAR (Table 2) which are substantially lower than the R^2^ values for model fits. These results raise the challenge of predicting the number of OFM trap catches in the subsequent year precisely. Instead, the respective R^2^ values for the short-term predictions were 0.592, 0.524, and <0 for the SARIMA-BIC, Prophet, and VAR (Table 2). These results inform that the short-term predictions should be practiced, rather than the long-term predictions. The results also imply that the VAR is not a reliable predictive model, and the SARIMA-BIC predicts one time point ahead as precisely as the Prophet.

The SARIMA-BIC selected SARIMA(0, 1, 1)(2, 0, 0)_8_ which utilized the non-seasonal parameters of *p* = 0 (no autoregressive terms to forecast the current value), *d* = 1 (differenced once for stationary), and *q* = 1 (using two previous error terms to forecast the current value) and the seasonal parameters of *p* = 2 (the current seasonal term is predicted by the two lagged seasonal values), *D* = 0, and *Q* = 0 (already stationary without differencing and seasonal moving average). It was surprising that this relatively simple time series structure outperformed the Prophet model, which allowed 25 potential changepoints by the default setting, for the short-term predictions of the national average number of OFM trap catches.

### 2.2. Province-Level Data (The Province of GB)

**Figure 3:**
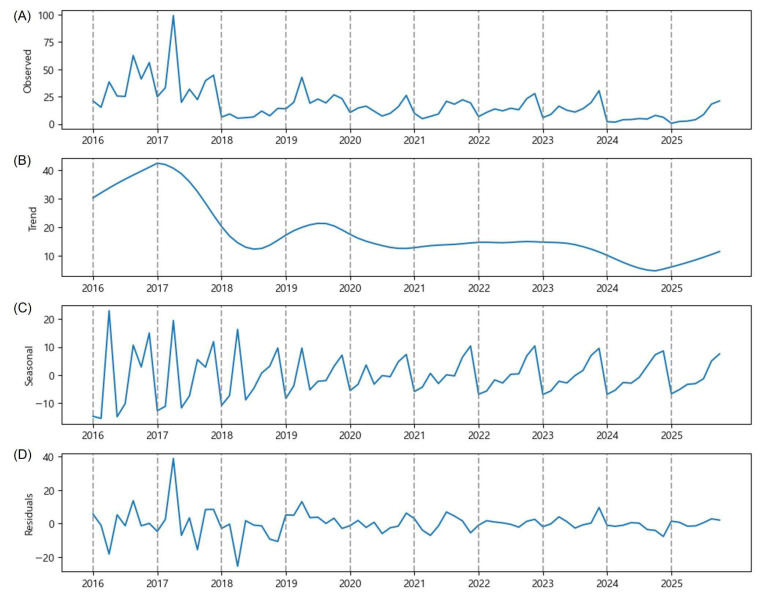
Time series decomposition of the number of OFM trap catches (y-axis) observed bimonthly between June and September from 2016 to 2025. They are the province-level data (GB): (**A**) the observed number of OFM trap catches, (**B**) estimated trend component, (**C**) estimated seasonal component, and (**D**) residuals.

In the province of GB, the most remarkable OFM outbreak occurred on 1 July 2017 (99.1 OFM trap catches), and it was the first OFM outbreak recorded in the Korean Peninsula since the data collection (Figure 3A). The following outbreak on 16 September 2017 was not as remarkable as the previous one, and the occurrence of OFM was suppressed in 2018. The number of OFM trap catches did not exceed 31 since then. The estimated trend is significantly downward after 1 July 2017, and it has been stable since 2018 (Figure 3B). The estimated seasonal component demonstrates three peaks in 2016 and 2017; two peaks from 2018 to 2021; and one peak since 2022 (Figure 3C). The magnitude of residuals has been stable since 2019 (Figure 3D).

Lee et al. [17] sampled apple orchards in Gunwi, a small district in the province of GB that consistently applied pesticides for pest management. They reported an annual total of 300 to 2000 OFM catches between 2006 and 2014, and it did not exceed 300 between 2015 and 2022. The decreasing trend of OFM is also shown in the province-level data (Figure 3B). Yang et al. [7] reported three or four peaks of OFM occurrence between late March and early October. As our data exclude March to May and early October, observing two or three peaks between June and September aligns with their study, and it appears that the natural life cycle of OFM has shifted over time to a single peak in September (Figure 3C).

**Figure 4:**
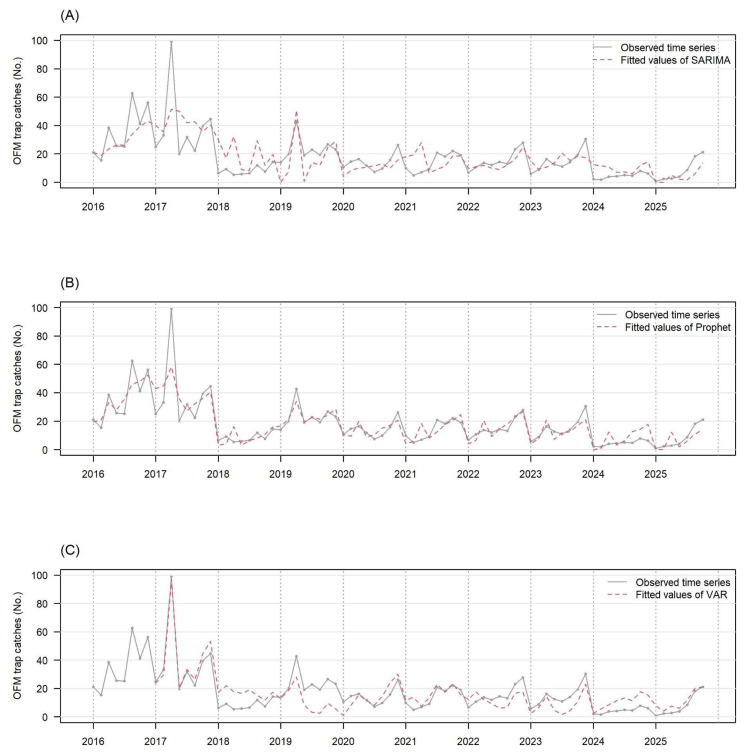
The observed number of OFM trap catches (gray solid line) and the fitted value by each time series model (red dotted line) are presented in this figure. They are the province-level data of GB. The fitted values are calculated by the SARIMA model (panel (**A**)), the Prophet model (panel (**B**)), and the VAR model (panel (**C**)). The VAR model needs observed data of the previous year for fitted values, so fitted values of 2016 are not available.

Figure 4B,C shows that the Prophet and VAR closely follow the observed time series data, whereas the SARIMA-BIC deviates from them as shown in Figure 4A. The respective R^2^ values for model fits (fitting all observed values from 2016 to 2025) were 0.455, 0.757, and 0.798 for the SARIMA-BIC, Prophet, and VAR. It appears that the observed values in the other provinces boosted the model fit of VAR, and its R^2^ value is slightly higher than the R^2^ value of the Prophet. The SARIMA-BIC selected SARIMA(0, 1, 1)(2, 0, 0)_8_ to fit the province-level data as well.

Supplementary Figure S2A–C shows the long-term predictions for the province of GB. The three models commonly overpredicted the outcomes in 2024 and underpredicted outcomes in 2025. The respective R^2^ values were 0.587, 0.448, and <0 for the SARIMA-BIC, Prophet, and VAR. Supplementary Figure S2D–F shows the short-term predictions for the province, and we can see that the VAR made multiple false alarms. The respective R^2^ values were 0.681, 0.564, and <0 for the SARIMA-BIC, Prophet, and VAR. The results imply that the short-term predictions of the SARIMA-BIC and Prophet were more reliable than the long-term predictions; the SARIMA-BIC predicted better than the Prophet even though its model fit (fitting all observed values from 2016 to 2025) was substantially worse than the Prophet; and it appears that time series data from the other provinces might be distractors when the VAR predicted long- and short-term future values.

### 2.3. Province-Level Data (The Province of JB)

**Figure 5:**
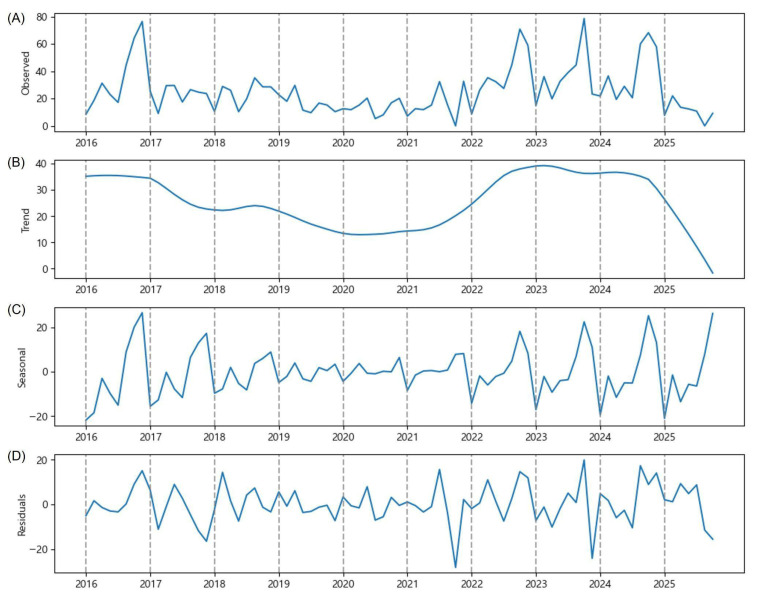
Time series decomposition of the number of OFM trap catches (y-axis) observed bimonthly between June and September from 2016 to 2025. They are the province-level data (JB): (**A**) the observed number of OFM trap catches, (**B**) estimated trend component, (**C**) estimated seasonal component, and (**D**) residuals.

Figure 5 provides the time series data of JB. The first remarkable OFM outbreak (76.5 OFM trap catches) was observed in 16 September 2016 (one year prior to the outbreak in GB); no remarkable peaks were observed between 2017 and 2021; and three remarkable peaks of 70.9, 78.7, and 68.3 were recorded in early September of 2022, 2023, and 2024, respectively (Figure 5A). Unlike the national average trend, the estimated trend in the province of JB is high from 2022 to 2024 (Figure 5B), and the estimated seasonal trend has shown two peaks, where the second peak is generally higher than the first peak within each year (Figure 5C). The magnitude of residuals is comparable to the magnitude of the seasonal component (Figure 5D) which challenges precise time series prediction.

**Figure 6:**
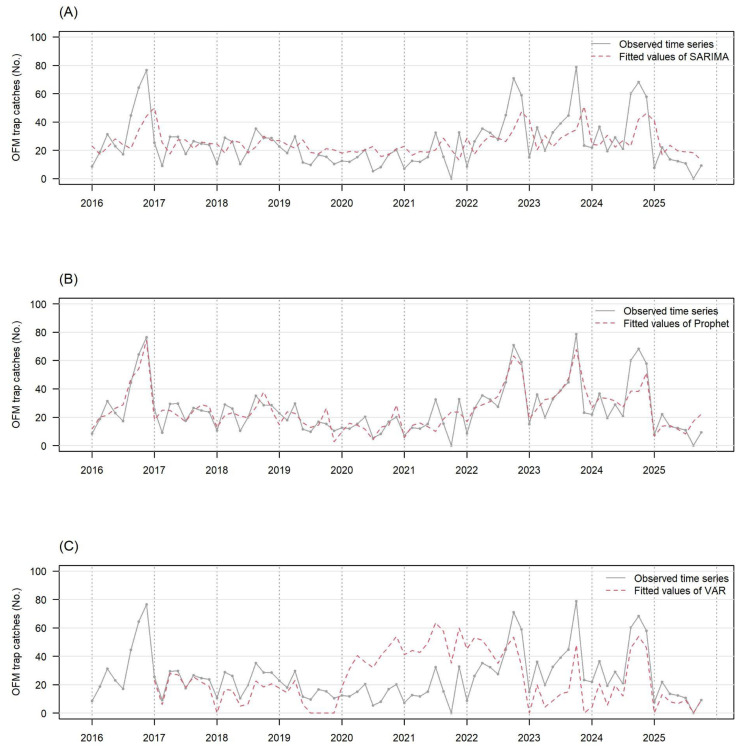
The observed number of OFM trap catches (gray solid line) and the fitted value by each time series model (red dotted line) are presented in this figure. They are the province-level data of JB. The fitted values are calculated by the SARIMA model (panel (**A**)), the Prophet model (panel (**B**)), and the VAR model (panel (**C**)). The VAR model needs observed data of the previous year for fitted values, so fitted values of 2016 are not available.

The Prophet provided a decent model fit with an R^2^ value of 0.739, and it accurately described three of the four major outbreaks in the last decade (Figure 6B). The fitted values of the VAR deviated substantially from the observed values (Figure 6C), and its overestimation from 2020 to 2022 stands out, followed by the underestimation in 2023 and 2024. After the three consecutive outbreaks from 2022 to 2024, all models overpredicted the outcomes in 2025 (Supplementary Figure S3A–C), and the short-term predictions of the VAR constantly overpredicted the first peak (Supplementary Figure S3F) which resulted in a negative R^2^ value. The time series data of JB were not as predictable as the time series data of GB (Table 3).

### 2.4. Province-Level Strategies Based on Short-Term Forecasts

**Figure 7:**
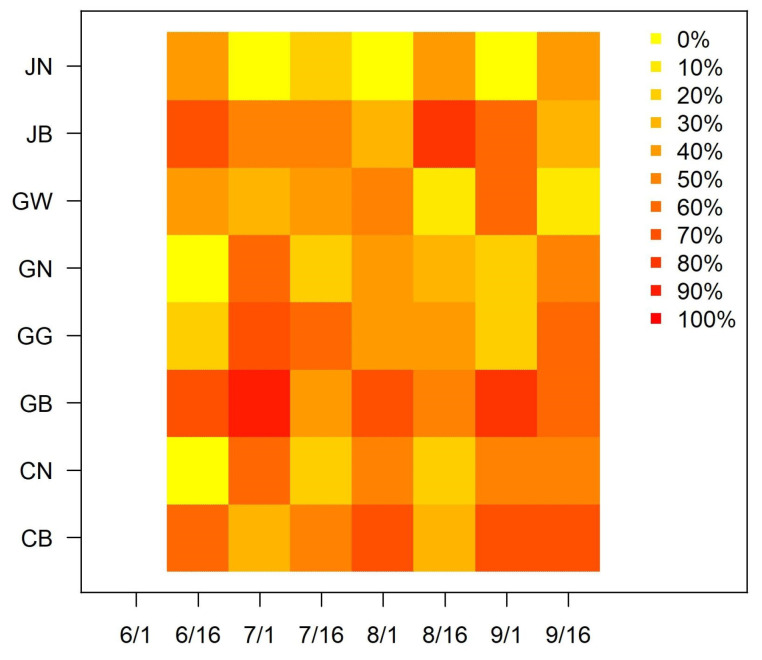
Relative frequency (% chance), estimated by the observed values from 2016 to 2025, that the observed OFM trap catches at a time point is greater than the observed OFM trap catches at the previous time point in each province.

Figure 7 graphically presents the likelihood of observing an increased number of OFM trap catches at a time point relative to the previous time point. The yellow color indicates the 0% chance for an increased number of OFM trap catches, the red color indicates the 100% chance, and figures between 0% and 100% are represented by the yellow to red gradient color scheme. The colors were determined based on all observed values from 2016 to 2025 for each province. The scattered colors imply that the upward and downward patterns are not uniform between the provinces, so it would be more practical to develop province-specific strategies for pest control.

As reported in Table 3, the short-term predictions for the four provinces (CB, GB, GN, and JB) are generally better than the long-term predictions. The short-term predictions of the SARIMA-BIC resulted in R^2^ values of 0.370, 0.681, 0.537, and 0.066 for CB, GB, GN, and JB, respectively, which are better than the respective long-term predictions (R^2^ values of <0, 0.587, 0.507, and <0 as reported in Table 1). Though the provinces of CB and GN have lower R^2^ values (0.370 and 0.537) than the province of GB (0.681), the provinces of CB and GN have lower RMSE values (2.958 and 2.071) than the province of GB (6.732) because the provinces of CB and GN have generally lower numbers of OFM trap catches than the province of GB. Therefore, the multiple measures should be evaluated together in the context.

The province of JN had mostly zero OFM trap catches (about 88% of the time before imputation), and the SARIMA-BIC and Prophet could forecast the low values with respective R^2^ values of >0.999 and 0.943 for short-term predictions and >0.999 and 0.965 for long-term predictions. The R^2^ value of the VAR was 0.594 for short-term predictions and 0.70 for long-term predictions. It is another demonstration that the VAR, which utilizes information from the other provinces, resulted in worse forecasting than the univariate time series models, the SARIMA and Prophet. The results suggest that the forecast is more accurate using its own province data, rather than using other provinces’ data as additional predictors, and practitioners should react to short-term forecasts (one time point ahead) rather than long-term forecasts (one year ahead), and this management system would require continually updated predictions like daily weather forecasts.

## 3. Discussion

The time series approach employed in this study is consistent with methodologies that have proven effective in pest management systems globally. The SOPRA forecasting system, developed for Switzerland fruit orchards, combines temperature-driven phenology models with field monitoring data to predict the development of codling moth (*Cydia pomonella*), *Grapholita lobarzewskii*, and six other major pests [19,20]. During several years, validation demonstrated that SOPRA reliably forecasts pest phenology under variable weather conditions, enabling growers to simulate based on local weather data, optimize spray timing and reduce unnecessary pesticide applications. The SOPRA was applied as a decision support system for the major insect pests of fruit orchards on a local and regional scale. Recent studies have also attempted to predict the seasonal population dynamics of OFM and *Adoxophyes orana* captured in sex pheromone traps installed within peach orchards in China [21]. Similarly, the PETE model, first developed in the 1970s for codling moth management in the United States, has been continuously refined and remains a standard tool for timing insecticide applications in Pacific Northwest apple orchards [10,11,18]. These systems share a common principle with our approach of utilizing sequential monitoring data to generate actionable forecasts that support pest management decisions.

More recently, time series and machine learning approaches have been applied to pest forecasting in diverse agricultural systems. A hybrid ARIMA–LSTM model developed for sugarcane pest and disease prediction demonstrated superior performance over standalone models, achieving lower prediction errors by capturing both linear trends and nonlinear fluctuations in pest incidence data [22]. In sub-Saharan Africa, the Pest Risk Information Service (PRISE) integrates weather data with phenological models to provide Short Message Service (SMS)-based early warnings to smallholder farmers, illustrating how forecasting systems can be scaled for widespread agricultural extension [23]. These examples underscore the growing recognition that predictive models, when integrated with monitoring infrastructure and communication platforms, can substantially improve pest management, which is our future direction.

Despite rapid warming and more frequent torrential rainfall on the Korean Peninsula, conditions favoring OFM populations, the national average number of OFM trap catches declined over the last decade (Figure 1B). The implementation of IPM reduced chemical pesticide use [24], while sex pheromone traps and mating disruptors have been widely used in the GB province. Moreover, drone-based surveillance has enhanced pest management [25]. These collective efforts are evidenced by the decreasing trend (Figure 3B) and the gradual transition from a double-peak to a single-peak seasonal pattern (Figure 3C), indicating that interventions have successfully disrupted the natural OFM life cycle.

The transition from a double-peak to a single-peak seasonal pattern observed in the national average and in the GB province (Figure 1C and Figure 3C) warrants deeper ecological interpretation. First, climate warming on the Korean Peninsula has accelerated the accumulation of growing degree-days (GDDs), which are the primary driver of OFM development and generation timing [3,17]. Lee et al. [17] documented rising temperature trends in major apple-growing regions of South Korea and reported the associated changes in pest occurrence patterns. Accelerated thermal accumulation may compress the temporal spacing between generations, causing previously distinct multi-generational peaks to merge into a single, broader peak later in the season. Second, the intensive implementation of IPM practices, particularly mating disruption and sex pheromone trapping, may have differentially suppressed early-generation populations (first and second generations). As OFM populations undergo exponential amplification across successive generations, the effective control of early generations can substantially reduce the magnitude of later peaks, potentially eliminating one or more seasonal peaks altogether.

Regarding the consistency of these shifts across years, our data indicate that the single-peak pattern in the GB province has been consistently observed since 2022 (three consecutive years), suggesting a structural shift rather than annual stochastic variation. In contrast, the JB province has maintained a consistent double-peak pattern throughout the study period, with the second peak (early September) being more pronounced than the first (early July) in most years (Figure 5C). This regional consistency suggests that the observed phenological patterns are stable within each province but differ between provinces, likely reflecting the differences in local climate and IPM implementation intensity as discussed above. These reasons explain the low predictive performance of the VAR model attempted in this study. Given that only three to four years of data are available since the apparent shift in 2022, continued long-term monitoring is necessary to confirm whether these patterns represent permanent structural changes or longer-term cyclical variations. A comprehensive review of the research findings suggests that, rather than relying solely on the time series model for predicting pest occurrence patterns, combining the time series model attempted in this study with GDD-based phenology models could serve as an alternative approach. The combination of the time series model and GDD-based model would enable more precise modeling of OFM phenology and provide stronger evidence for the ecological drivers of these peak shifts in the OFM population.

The findings of this study have direct implications for operational pest management in apple orchards. The superior performance of short-term predictions (R^2^ = 0.592 for SARIMA-BIC at the national level) compared to long-term predictions (R^2^ = 0.177) suggests that a dynamic forecasting approach, where models are updated with each new observation, is more suitable for practical decision support than static annual forecasts. In practical terms, the short-term forecasting framework evaluated in this study can be integrated into an early warning system that provides biweekly alerts to agricultural officers and farmers. When the predicted number of OFM trap catches exceeds a predetermined economic threshold, the system can issue recommendations for intensified monitoring or targeted pesticide applications. For instance, in the JB province, where consecutive outbreaks were observed from 2022 to 2024, such a system would have detected the escalating trend in 2022 (Figure 5C) and could have prompted preemptive management actions before the outbreak intensified.

Furthermore, the regional variation in model performance highlights the need for province-specific management strategies. The successful suppression of OFM in tGB province, evidenced by the declining trend and transition from double-peak to single-peak seasonal patterns, demonstrates the effectiveness of intensive IPM practices including sex pheromone-based mating disruption and drone-assisted surveillance [24,25]. Extending these proven strategies to the JB province, where OFM populations remain problematic, could be prioritized based on our predictive model outputs.

In South Korea, the Rural Development Administration (RDA) undertakes government-level agricultural activities, and its affiliated agencies lead the surveillance and monitoring of fruit tree pests including OFM. To fully benefit from short-term predictions, the RDA officials should establish the foundations of more complete, precise, and frequent sampling than the current practice. Currently, five apple orchards are sampled per city due to limited resources and labor, and we need more trained investigators to improve the predictive performance, and hence the usefulness, of the time series models. As an initial roadmap, we encourage pilot trials in a few selected provinces and sample orchard clusters at least weekly during the critical season, analyzing orchard-level data instead of province-level aggregated data, and integrating local microclimatic data to accurately understand and estimate their association with outbreaks.

In this study, the VAR model performed poorly in both long- and short-term predictions. These results suggest weak correlations among the province-level aggregated data, where data from other provinces acted as distractors rather than predictors in the multivariate time series modeling. If local data are available, and if neighboring orchards show similar patterns or lagged patterns, the VAR model still can be a good candidate. Instead, the SARIMA model (with the BIC) provided the relatively reliable long- and short-term predictions at the province-level (Table 2 and Table 3), despite showing a poorer model fit to the entire time series data than the Prophet and VAR models (Table 1). That is, the simpler time series model structure was preferred for the available data in this study.

It should be noted that the province-level comparisons in this study are subject to several potential confounding factors that may influence the observed patterns independently of the model structure. First, climatic conditions vary substantially between provinces. The inland areas of the GB province are characterized by many basin-like topographies, resulting in large diurnal temperature variations. The west coast region of the JB province experiences a relatively milder climate than inland areas at the same latitude due to the influence of the sea. These differences affect the accumulation of degree-days, which is a critical determinant of OFM phenology and generation timing [17]. Second, the level of IPM implementation varies between provinces. As discussed earlier, the GB province has actively adopted advanced pest management technologies, including drone-based surveillance and mating disruption systems, which may have contributed to the declining trend and single-peak seasonal pattern observed in recent years (Figure 3C). In contrast, the consecutive outbreaks observed in the JB province from 2022 to 2024 may reflect differences in the intensity or timing of pest management interventions. Therefore, the differences in model performance between provinces (higher R^2^ values for GB compared to JB in short-term predictions; Table 3) should be interpreted with caution, as they may partially reflect these environmental and management heterogeneities rather than purely methodological factors.

Our ultimate goal is to establish a Smart IPM system capable of monitoring, predicting, and managing fruit tree pests and diseases in the nation. However, several limitations of this study should be acknowledged to improve short-term predictions. First, some provinces were removed in the analysis due to missing data, and scarce and incomplete data collection is our current challenge. Currently, five apple orchards are sampled per city due to limited resources and labor, and more trained investigators and frequent surveys are needed to improve the predictive performance and practical usefulness of the time series models. Second, sex pheromone trap catch data have inherent biases that may affect the interpretation of our results. Pheromone traps selectively attract male adults. Therefore, trap catches do not directly reflect the total population size or female abundance. Additionally, trap efficiency is influenced by several meteorological conditions, including temperature, relative humidity, and wind speed, which affect male flight activity [26,27]. Under identical population densities, trap catches can vary substantially depending on the weather conditions. Third, it is important to recognize that forecasting trap catches is not equivalent to forecasting damage risk. Trap catches reflect the flight activity of male adults, whereas economic damage is caused by larval feeding on fruit. The relationship between male adult density captured in traps and subsequent fruit damage involves multiple ecological processes, including mating success, female oviposition rates, and egg and larval survival. The time series models attempted in this study are based on the assumption that fruit damage increases proportionally to the number of male adults captured in traps. While these time series models can forecast the relative magnitude and outbreak timing of OFM populations, translating these predictions into fruit damage risk assessments requires additional research integrating trap catch data with fruit damage surveys, larval monitoring, and environmental covariates. Future studies should aim to develop predictive models that directly link trap catches to economic damage thresholds, thereby providing actionable guidelines to support timely decision-making for pest and disease control at the local level. Despite these limitations, this study demonstrated both the potential and challenges of time series forecasting for OFM management, and we hope that these findings will accelerate progress toward a comprehensive Smart IPM system.

## 4. Materials and Methods

### 4.1. Data

The OFM data (the number of OFM trap catches) were provided by the National Crop Pest Management System (NCPMS, https://ncpms.rda.go.kr, accessed on 1 December 2025) managed by the RDA. There are eight administrative districts (provinces) in South Korea including Gyeonggi-do (GG), Gangwon-do (GW), Chungcheongbuk-do (CB), Chungcheongnam-do (CN), Jeollabuk-do (JB), Jeollanam-do (JN), Gyeongsangbuk-do (GB), Gyeongsangnam-do (GN). The agricultural technology centers located in these provinces annually collect data on the occurrence of pests and diseases affecting rice, apples, pears, peaches, peppers, onions, and garlic. This study focused on pests affecting apple trees, and the data were collected in the following cities where apples are widely cultivated: Gapyeong and Pocheon in GG; Yeongwol and Chuncheon in GW; Boeun, Yeongdong, Jecheon, and Chungju in CB; Dangjin and Yesan in CN; Muju and Jeongeup in JB; Jangseong in JN; Sangju, Andong, Yeongju, Yeongcheon, Uiseong, Cheongsong, and Pohang in GB; and Geochang and Miryang in GN.

A new cohort of 5 apple orchards is sampled annually per city in each province. For example, the province of GG samples 10 new orchards annually in the cities of Gapyeong and Pocheon. An official investigator from the agricultural technology center in each city conducts pest monitoring. That is, the province of GG conducts pest monitoring in 10 new orchards (5 in Gapyeong and 5 in Pocheon) by 2 investigators every year. Similarly, the provinces of GW, CB, CN, JN, JN, GB, and GN sample 10, 20, 10, 10, 5, 35, and 10 new orchards, respectively, and data are collected by 2, 4, 2, 2, 1, 7, and 2 investigators, respectively. A total of 22 investigators, who have a university degree in horticulture and are trained by the RDA on fruit tree pests and diseases on a regular basis, conduct pest monitoring on 110 apple orchards sampled across the nation every year, and they follow the same protocol. Pheromone traps are installed in local orchards, and they count the number of OFM captured in the traps. The numbers estimated across cities are aggregated and averaged in each province. The NCPMS have published the average values in each province on the first day and the sixteenth day of June, July, August, and September (i.e., 8 times for the 4-month period) since 2016. For this study, we obtained all values from 2016 to 2025, except the last data point (16 September 2025) which was not available at the time of data analysis.

Sex pheromone traps (Delta trap, Greenagrotech, Gyeongsan, Korea) were installed in local orchards, by hanging them at a height of 1.5 m on the apple tree canopy, and the lure was a combination of three sex pheromone components, (Z)-8-dodecenyl acetate, (E)-8-dodecenyl acetate, and (Z)-8-dodecenol, in a ratio of 88.5:5.7:1.0 for OFM in apple orchards [17]. The distance between traps was set to at least 10 m to minimize interference effects between pheromone traps, and the sticky boards in traps were replaced every 10 days, and the lures were replaced every two months [17]. The location (latitude and longitude) and area data for each orchard can be downloaded via the open API provided by NCPMS. Downloading and utilizing these data is restricted to Korean nationals only.

Based on our preliminary analysis, about 50% of the variation in the number of OFM trap catches was explained by the province, and the provinces of GB and JB accounted for 70% of the national number of OFM trap catches. The provinces of CN, GW, and JN had zero or very low numbers of OFM trap catches as apples are not widely cultivated in these regions, so clear seasonal patterns could not be observed in the time series data. There were some missing values in the provinces of GG, GW, and JN (23%, 46%, and 28% were missing, respectively). Due to these limitations, we present the results on the national average and the two specific provinces, GB and JB, in detail, and the results of two additional provinces, CB and GN, are briefly reported. The Prophet model (Section 4.2) which fitted the observed data the best was used to impute missing values in the provinces. The weighted average was used to estimate the national average number of OFM trap catches, where the weights were determined proportional to the number of orchards sampled per province.

All surveys were conducted after the consent of orchard owners. According to the Act on Promotion of the Provision and Use of Public Data, all South Korean citizens have the right to access and utilize the public data. The open data are publicly available and accessible to South Korean citizens and are allowed for research purposes.

### 4.2. Statistical Analysis

Three time series models were considered: the seasonal autoregressive integrated moving average (SARIMA) model, the Prophet model, and the vector autoregressive (VAR) model with both a constant and a time trend component.

The SARIMA model is an extension of the ARIMA model, and it is devised to capture seasonality and trends of time series data in addition to the non-seasonal components that ARIMA captures [28]. The forecast package was used in R for fitting the SARIMA model [29,30]. The default parameter values were set in the auto.arima function with the seasonality option, and it searched for the best parameter values based on one of the following information criteria: Akaike Information Criterion (AIC), AIC with a correction (AICc), and Bayesian Information Criterion (BIC).

The Prophet model, developed by Facebook in 2018, is another popular time series model which automatically detects trend changes, and it is known to perform well with strong seasonal effects [31]. Its algorithm is based on an additive regression model, which consists of a trend function modeling non-periodic changes, a seasonality function modeling periodic changes, a holiday effect modeling irregular schedule effects (which is not relevant for data in this study), and the error term unexplained by the model. The Prophet package was used in R for fitting the Prophet model [32]. The default tuning parameter values were used with annual seasonality and without weekly and daily seasonality and holiday effects.

The VAR model is a time series model for multiple time series data, which are potentially correlated, and it is useful when the current data point of a time series can be predicted by past data point(s) of the time series and of other time series [33]. The VAR model is often used for economic time series data for various purposes [34], and it is applied in this study for describing observed OFM data and forecasting future OFM data. For describing the national average of OFM trap catches, the time series data of all eight provinces are modeled, and the weighted average of province-level fitted or predicted values was used to determine the national average number of OFM trap catches, where the weights were determined proportional to the number of orchards sampled per province. The var and tseries packages were used in R for fitting the VAR model and conducting the Augmented Dickey–Fuller (ADF) test [35,36], and the data were differenced once for stationary according to the ADF test.

The SARIMA model is known to capture stable regular patterns well, and the Prophet model is known to detect shifts in trends in addition to the advantage of a traditional time series model [37]. The VAR model borrows information from other provinces; hence, it may provide more accurate predictions if there are strong correlations of the number of OFM trap catches across the provinces. Otherwise, the VAR model may underperform due to the distracting information.

The quality of model fit and long- and short-term predictions was measured by the mean absolute error (MAE), root mean square error (RMSE), and R-square (R^2^). Let MSE_0_ denote the mean square error (MSE) of the null model, which fits all time series data points by the simple average. Given the MSE of a time series model, the R-square value is calculated as R^2^ = 1 − MSE/MSE_0_. When a time series model predicts future data values worse than the null model, it is possible to observe R^2^ < 0 [38,39]. A negative R^2^ value implies that the time series pattern is very unpredictable and/or the time series model provides very unreliable predictions (worse than guessing by the simple average of the entire time series).

Both long- and short-term predictions were evaluated by moving the time window forward from 2021 to 2025. For long-term predictions, each model was trained by time series data until year *m* − 1 to predict 8 data points in year *m* for *m* = 2021, 2022, 2023, 2024 and 7 data points in year 2025 as the data point of 16 September 2025 was not available at the time of analysis. For instance, the 8 data points of the year 2021 were simultaneously predicted by training each model using data points up to the year 2020; the 8 data points of the year 2022 were predicted by training each model using data points up to the year 2021; and so on until predicting the 8 data points of the year 2025 using data points up to the year 2024. For short-term prediction, each model was trained by time point *n* − 1 to predict the subsequent time point *n* for 1 June 2021, 16 June 2021, …, 1 September 2025. For instance, the data point of 1 June 2021 was predicted by training each model using data points up to 16 September 2020; the data point of 16 June 2021 was predicted using data points up to 1 June 2021; and so on until predicting the data point of 1 September 2025 using data points up to 16 August 2025. Both long- and short-term forward-chaining predictions were evaluated by the MAE, RMSE, and R^2^ by comparing the predicted values and the actual observed values from 2021 to 2025. All statistical analyses and data visualizations were performed in R Version 4.5.1 [40].

According to the current data collection schedule described in Section 4.1, the long-term prediction means forecasting about nine months ahead, and the short-term prediction means forecasting about two weeks ahead. If the predictions are accurate, the long-term forecasting will help when planning pest management ahead of the following season, and short-term forecasting will help when reacting to an unexpected OFM occurrence or outbreak in a timely manner. Therefore, evaluations of both short- and long-term predictions are important. The time series models provide a stable early signal when the annual trend is stable (constant or gradually increasing or decreasing) with strong seasonal patterns (repeating over years) and a relatively small magnitude of random noise. A time series model generally makes a large long-term prediction error when a sudden change (e.g., an unexpected outbreak) occurs, and a short-term prediction will be helpful as it reacts to the sudden change and makes a signal for an immediate action.

## 5. Conclusions

The time series analysis revealed the trend and seasonal components of the number of OFM trap catches in apple orchards, and it demonstrated a downward trend along the transition from double-peak to single-peak seasonal patterns. It reflected the effect of pest management for the last decade in most regions in South Korea, especially the advanced drone-based surveillance and conventional mating disruptors in the GB province. This study presented more reliable OFM population forecasts by utilizing short-term predictions rather than long-term predictions, and demonstrated that the multivariate time series modeling did not improve the forecasts with province-level aggregated data. The usefulness of time series models can be leveraged by orchard-level data reporting, frequent sampling, pest management records, and the incorporation of precise environmental information. These collective efforts will support actionable decisions for protecting fruit quality and increasing yield.

## Figures and Tables

**Table 1:** Evaluations of the model fits (using all observed values) of the SARIMA (AIC, AICc, and BIC), Prophet, and VAR models.

Metric	Region	SARIMA (AIC)	SARIMA (AICc)	SARIMA (BIC)	Prophet	VAR
MAE	CB	1.955	1.955	1.955	1.447	1.692
	GB	7.798	7.798	7.897	5.052	5.657
	GN	1.551	1.551	1.654	1.074	1.385
	JB	11.551	11.551	11.626	6.524	13.162
	National	3.111	3.111	3.111	1.819	2.800
RMSE	CB	2.708	2.708	2.708	1.919	2.316
	GB	10.943	10.943	11.336	7.573	6.901
	GN	2.246	2.246	2.410	1.497	1.912
	JB	14.712	14.712	15.106	8.813	16.312
	National	4.439	4.439	4.439	2.621	3.495
R^2^	CB	0.411	0.411	0.411	0.704	0.569
	GB	0.492	0.492	0.455	0.757	0.798
	GN	0.413	0.413	0.325	0.739	0.575
	JB	0.273	0.273	0.233	0.739	0.106
	National	0.411	0.411	0.411	0.795	0.635

**Table 2:** Evaluation of the long-term predictions of the SARIMA (AIC, AICc, and BIC), Prophet, and VAR models.

Metric	Region	SARIMA (AIC)	SARIMA (AICc)	SARIMA (BIC)	Prophet	VAR
MAE	CB	3.175	3.227	3.348	4.436	5.381
	GB	10.052	9.986	6.578	7.217	12.391
	GN	1.640	1.604	1.692	1.758	2.197
	JB	19.932	19.932	19.932	19.248	37.372
	National	4.410	4.173	3.849	3.542	7.116
RMSE	CB	3.951	3.975	4.087	5.710	6.593
	GB	11.701	11.956	7.920	9.154	15.259
	GN	2.279	2.223	2.229	2.839	3.001
	JB	24.814	24.814	24.814	24.221	55.417
	National	5.159	4.986	4.730	4.409	8.445
R^2^	CB	<0	<0	<0	<0	<0
	GB	0.099	0.059	0.587	0.448	<0
	GN	0.485	0.510	0.507	0.200	0.106
	JB	<0	<0	<0	<0	<0
	National	0.021	0.085	0.177	0.285	<0

**Table 3:** Evaluations of the short-term predictions of the SARIMA (AIC, AICc, and BIC), Prophet, and VAR models.

Metric	Region	SARIMA (AIC)	SARIMA (AICc)	SARIMA (BIC)	Prophet	VAR
MAE	CB	2.150	2.171	2.262	3.347	2.512
	GB	6.924	6.891	5.526	6.317	13.215
	GN	1.455	1.463	1.479	1.360	2.014
	JB	15.831	15.781	15.887	14.175	23.400
	National	3.084	2.817	2.422	2.904	5.707
RMSE	CB	2.997	2.913	2.958	4.087	3.323
	GB	8.179	8.190	6.732	7.871	16.847
	GN	2.085	2.090	2.071	2.249	3.233
	JB	19.066	19.018	19.568	17.138	35.185
	National	3.788	3.619	3.210	3.465	7.387
R^2^	CB	0.353	0.389	0.370	<0	0.205
	GB	0.529	0.528	0.681	0.564	<0
	GN	0.531	0.529	0.537	0.454	<0
	JB	0.113	0.118	0.066	0.284	<0
	National	0.432	0.481	0.592	0.524	<0

## Data Availability

Publicly available datasets were analyzed in this study. These data can be found here: https://ncpms.rda.go.kr.

## Supplementary Materials

The following supporting information can be downloaded at https://www.mdpi.com/article/10.3390/plants15040624/s1, Figure S1: The observed time series and the long-term and short-term predictions by the SARIMA, Prophet, and VAR models (the national average data). Figure S2: The observed time series and the long-term and short-term predictions by the SARIMA, Prophet, and VAR models (the GB province data). Figure S3: The observed time series and the long-term and short-term predictions by the SARIMA, Prophet, and VAR models (the JB province data).
