# Supplementary material for: Long-Term and Short-Term Forecasting of Oriental Fruit Moth (Grapholita molesta) Trap Catches from Apple Orchards in South Korea Using Time Series Models
Source: Plants (Basel). 2026 Feb 16;15(4):624. doi: 10.3390/plants15040624 (PMC12943878; doi:10.3390/plants15040624)
Supplement: Supplementary file 1 [file plants-15-00624-s001.zip › plants-4105298-supplementary.pdf]

Supplementary Figures

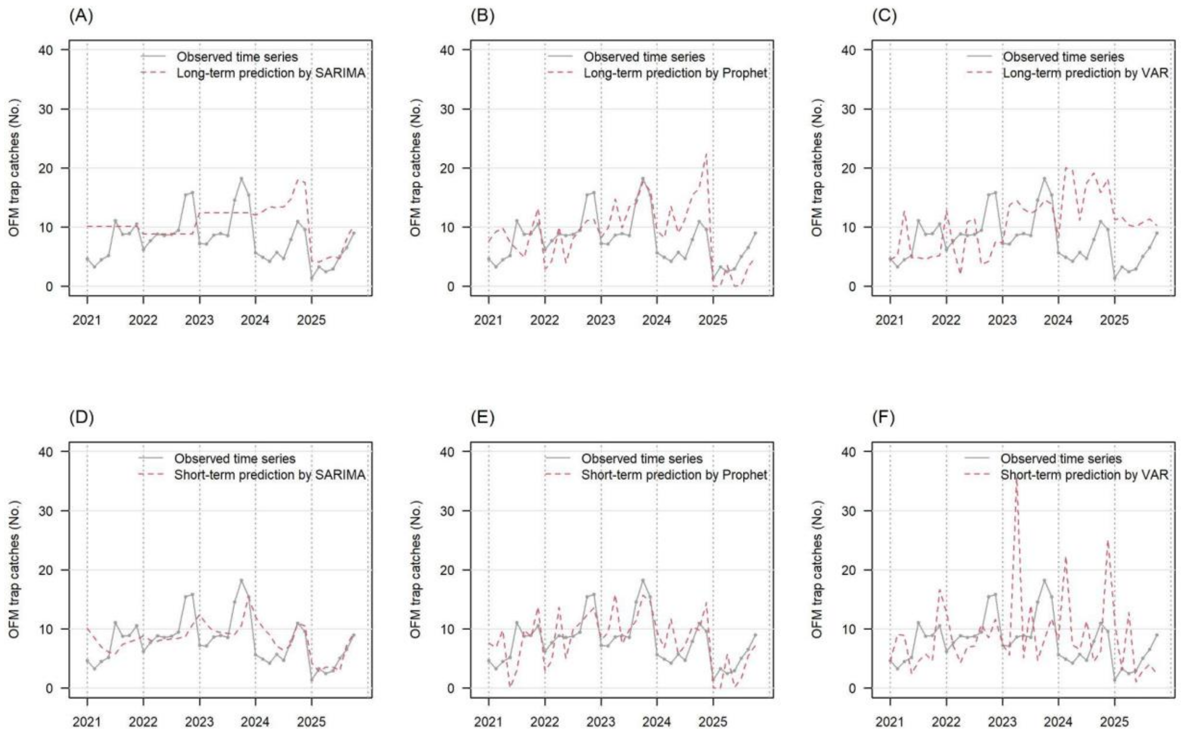

Figure S1. The observed number of OFM trap catches (grey solid line) and the predicted value by each time series model (red dotted line) are presented in this figure. It is the national average data. The long-term predictions are performed by the SARIMA model (panel A), the Prophet model (panel B), and the VAR model (panel C); and the short-term predictions are performed by the SARIMA model (panel D), the Prophet model (panel E), and the VAR model (panel F).

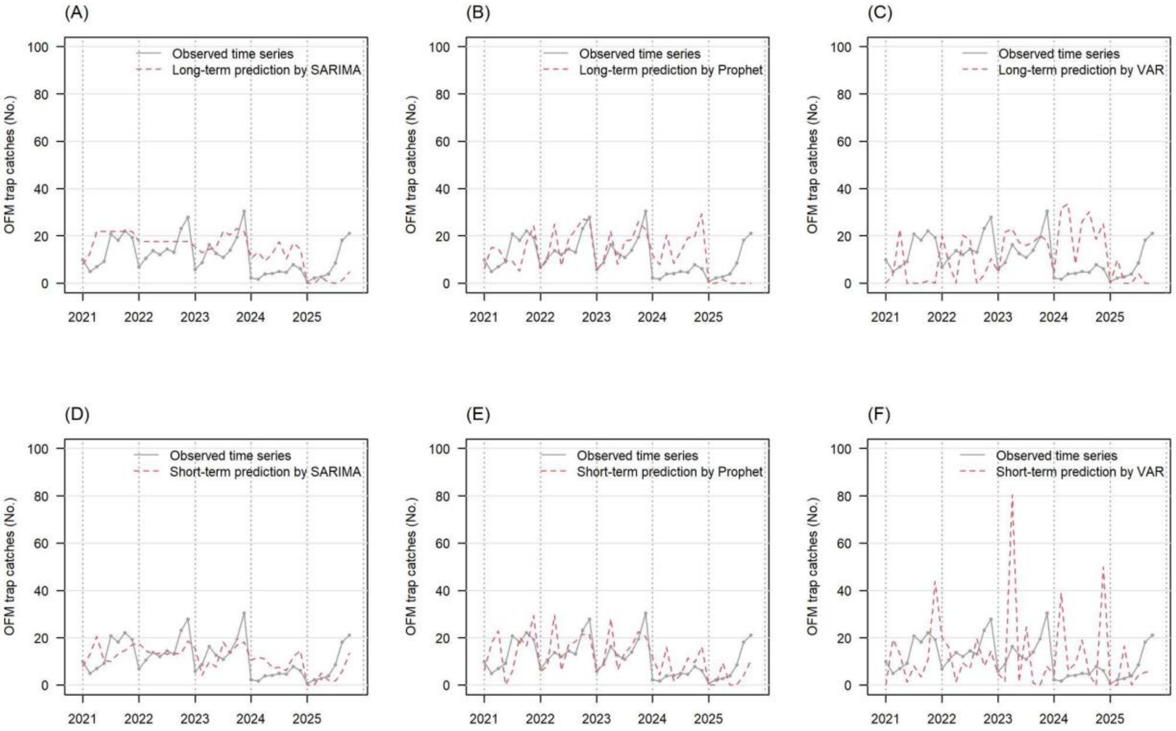

Figure S2. The observed number of OFM trap catches (grey solid line) and the predicted value by each time series model (red dotted line) are presented in this figure. It is the province-level data of GB. The long-term predictions are performed by the SARIMA model (panel A), the Prophet model (panel B), and the VAR model (panel C); and the short-term predictions are performed by the SARIMA model (panel D), the Prophet model (panel E), and the VAR model (panel F).

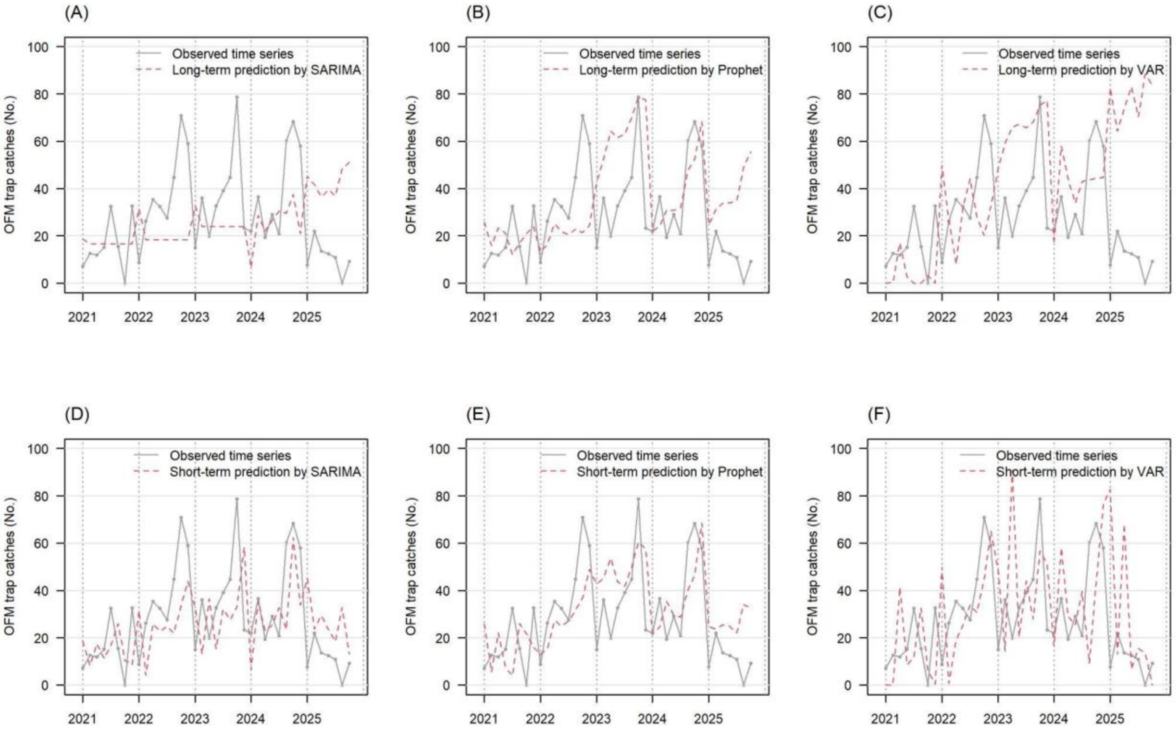

Figure S3. The observed number of OFM trap catches (grey solid line) and the predicted value by each time series model (red dotted line) are presented in this figure. It is the province-level data of JB. The long-term predictions are performed by the SARIMA model (panel A), the Prophet model (panel B), and the VAR model (panel C); and the short-term predictions are performed by the SARIMA model (panel D), the Prophet model (panel E), and the VAR model (panel F).
